# Evaluation of ChatGPT’s reliability in answering questions about short stature and growth failure

**DOI:** 10.1016/j.jped.2026.101577

**Published:** 2026-07-02

**Authors:** Rebeca Cricca Avancini, Carlos Alberto Longui

**Affiliations:** Faculdade de Ciências Médicas da Santa Casa de São Paulo, SP, Brazil

**Keywords:** AI, ChatGPT, Pediatric endocrinology, Short stature

## Abstract

**Objective:**

To evaluate ChatGPT’s performance in responding to questions related to short stature and growth failure, which are common concerns in clinical practice.

**Methods:**

Answers provided by the GPT-4 and GPT-4o models to a 36-item questionnaire administered at three different time points (baseline, after 3 months, and after 1 year) were assessed. Each response was rated on a 0–6 Likert scale according to their agreement with those given by pediatric endocrinology specialists.

**Results:**

ChatGPT showed an overall satisfactory performance, with high agreement in conceptual questions and in respect of common causes, but lower accuracy in diagnostic and therapeutic topics that require more specialized knowledge. The mean score was 4.6 (SD = 1.3), with a mode of 6. Using a threshold of scores 3–6 as correct, 96.9% of responses were classified as satisfactory; applying stricter thresholds of scores 4–6 and 5–6 yielded rates of 77.2% and 46.3%, respectively. An improvement was observed after the update to GPT-4o, suggesting model evolution over time. However, there was a significant decrease in recommendations to seek medical care in the more recent model.

**Conclusion:**

ChatGPT demonstrated high reliability and potential as a supportive tool for issues related to short stature; however, its lower accuracy in therapeutic topics and reduced emphasis on the importance of seeking professional medical advice highlight the need for further improvements to ensure its safe use in healthcare. Both professionals and lay users be aware of the limitations of AI and verify information through reliable medical sources.

## Introduction

ChatGPT is a chatbot developed by the American company OpenAI based on the Generative Pre-trained Transformer (GPT) language system [1]. Launched in November 2022, it quickly gained millions of users due to its ability to simulate human-like conversations. Since then, it has undergone major updates, including GPT-4 in 2023 and GPT-4o in 2024, which expanded its functions to include real-time interpretation of text, images, and audio [2]. By July 2025, it already had around 122 million daily users, demonstrating its global popularity [3]. Despite its success, the tool still presents limitations, which are acknowledged by OpenAI itself. Among these are “hallucinations”, incorrect but convincing responses, as well as difficulties in understanding humor, metaphors, and subjectivity. Even GPT-4, which is considered 40% more accurate than previous versions, may still provide incorrect or outdated information, since the models do not have access to the internet in real time [4].

ChatGPT has broad applicability in different fields, such as programming, translation, and text editing [5]. In medicine, it has shown remarkable performance in standardized tests such as the United States Medical Licensing Examination (USMLE): ChatGPT (GPT-3.5) performed at or near the passing threshold, while GPT-4 achieved an overall accuracy of approximately 81%, outperforming the average scores of medical students in multiple studies [6]. These findings highlight the potential of AI in supporting diagnosis and clinical decision-making, although experts emphasize the need for further studies to validate its safety and effectiveness.

In the context of digital health, there has been a steady rise in “e-patients”, individuals who seek medical information online before consulting a doctor. This behavior can contribute to the spread of inaccurate beliefs and increase physicians’ workload during consultations [7]. While traditional online symptom checkers such as Google have shown low accuracy [8], ChatGPT has emerged as a promising alternative for health information seeking, with studies consistently demonstrating its potential to provide high-quality responses to patient and clinician questions across a variety of medical domains [5]. Its application has been explored specifically in pediatric endocrinology: a study evaluating ChatGPT and other Large Language Models (LLMs) in response to questions about type 1 diabetes in children found generally accurate and comprehensible answers, though performance varied by topic complexity [9]; similarly, an evaluation of ChatGPT responses to questions on pediatric endocrine and metabolic conditions, including short stature, across Chinese and English-language contexts found that performance differed by language and by the clinical domain assessed [10]. Its application could be particularly relevant in specialized areas such as pediatric endocrinology, especially in countries like Brazil, where a shortage of professionals often limits access to expert guidance [11]. One of the most frequent concerns in this field is short stature [12], diagnosed when a child’s height on the general population growth curve falls below the 2.5th percentile (or −2 standard deviations) [13]. It may arise from both endocrine and non-endocrine causes, ranging from genetic syndromes to chronic systemic diseases, each with distinct and complex pathophysiological mechanisms, diagnostic methods, and therapeutic indications [13].

Thus, this study aimed to evaluate the reliability of GPT’s responses to questions related to short stature and growth failure by comparing them with those given by experts in the field.

## Materials and methods

This was a comparative agreement study, in which the responses provided by the GPT-4 and GPT-4o models were compared with answers from experts in pediatric endocrinology. The objective was to assess the reliability of GPT-4 and GPT-4o responses to questions related to short stature and growth failure. In addition, the study aimed to identify the evolution of GPT’s answers after 3 months and 1 year, as well as to analyze the pattern of recommendations for seeking medical care given to users who asked questions on this topic.

### Questionnaire development

A 36-item questionnaire was developed, covering both medical questions (focused on diagnosis and management) and layperson-oriented questions (simpler content representing the most common doubts expressed by patients and their families during medical consultations).

The full questionnaire, including all questions and expected answers provided by the specialists, is available in the Supplementary Material.

The topics were divided into five thematic areas:

• Definitions and Concepts
• Common Causes
• Diagnostic Methods
• Therapeutics
• Disease or Treatment Outcomes

The questions addressed the main causes of short stature and growth deficiency.

### Questionnaire application

Each question was submitted individually to ChatGPT-4 three times in sequence, with variations in phrasing to assess consistency. The responses generated by the program were stored for later analysis. The same procedure was repeated after three months (GPT-4) and one year (GPT-4o).

### Evaluation of responses

All chatbot responses were independently assessed by three pediatric endocrinology specialists. Each evaluator rated the responses blinded to the time point and model version of each chatbot response. Evaluators worked independently, without knowledge of the other evaluators’ scores. In cases of disagreement, consensus was reached through discussion and alignment. The expected reference responses were developed based on established guidelines and current medical literature, specifically the *Endocrinologia para o pediatra.* 3rd edition [13] and the *Endocrinologia Pediátrica: Guia Prático*, 4th edição [14]. The chatbot responses were assessed using a Likert-type scale ranging from 0 to 6:

• 0: Completely incorrect.
• 1: Incorrect but containing partial factual information.
• 2: Correct but containing inaccurate details.
• 3: Correct but incomplete.
• 4: Correct and complete but lacking clarity and/or appropriateness of language for a medical or lay audience.
• 5: Completely correct and complete.
• 6: Completely correct and more comprehensive than the reference answer.

This evaluation was performed in the same manner for the first, second, and third questionnaire applications.

### Statistical analysis

The scores assigned to each chatbot response were compiled and tabulated for statistical evaluation. Descriptive analyses were conducted to assess the chatbot’s overall performance, including calculations of the mode, mean, median, standard deviation, and interquartile range, in order to determine the level of agreement between ChatGPT’s responses and the reference answers established by pediatric endocrinology specialists. Each question category and each questionnaire application were analyzed individually to explore variations in performance across domains and over time. Comparisons were made between the different thematic areas to assess whether the chatbot performed differently depending on the topic, and between successive questionnaire applications to identify potential improvements in theoretical knowledge or consistency of responses. Finally, the analysis also verified whether ChatGPT recommended that users seek medical assistance when appropriate.

## Results

Of the 324 responses provided by the chatbot across the three applications, 44.4% received a score of 6, 1.8% a score of 5, 30.8% a score of 4, 19.7% a score of 3, 2.7% a score of 2, and 0.3% a score of 1. The mean score was 4.6, the median was 4.0, the mode was 6.0, and the standard deviation observed was 1.3.

**Figure 1:**
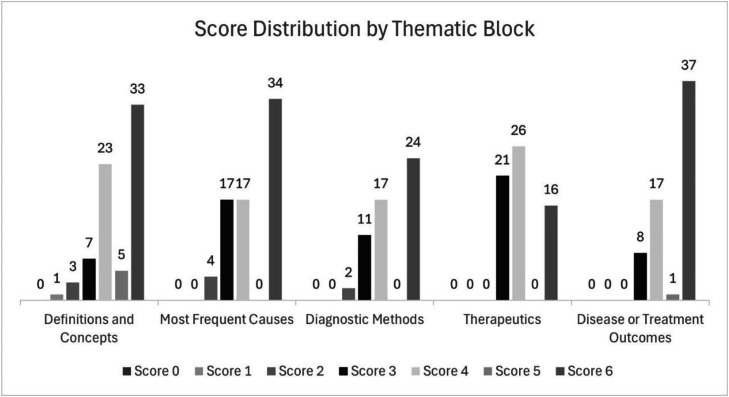
Score distribution by thematic block.

As illustrated in Figure 1, performance varied across different thematic domains, being more consistent in questions related to definitions, concepts, and most frequent causes, with a predominance of completely correct and complete answers. The disease or treatment outcomes domain yielded the greatest concentration of higher scores, suggesting better performance in questions of a descriptive or conceptual nature. In contrast, the diagnostic methods and therapeutics domains showed greater variability in scores, indicating that the model had more difficulty with topics requiring clinical reasoning and specialized knowledge. These results demonstrate the chatbot’s solid understanding of theoretical topics while also highlighting its limitations in practical and decision-making aspects of medicine.

When comparing the first and second applications, both using ChatGPT-4, of the 108 questions analyzed, 71.3% remained the same, 20.4% showed a decrease in score and 8.3% improved. When comparing the first and third application, which used GPT-4o, 48.1% maintained the same score, 18.5% had a lower score and 33.3% achieved a higher score. When the responses were classified into two groups: scores 1–3 and scores 4–6, and the chatbot’s initial and final performance were assessed, 70.4% remained in the same category, 19.4% improved, and 10.2% worsened, as shown in Table 1. Although these differences were not statistically significant (p = 0.110) power analysis based on the Chi-square test for proportions indicated that if the same response pattern was maintained, statistical significance would be reached with 283 questions.

**Table 1:** Comparison between initial and final applications.

		Final Application		
		1 to 3	4 to 6	Total
**Initial Application**	**1 to 3**	5	21	26
	**4 to 6**	11	71	82
	**Total**	16	92	108

**Figure 2:**
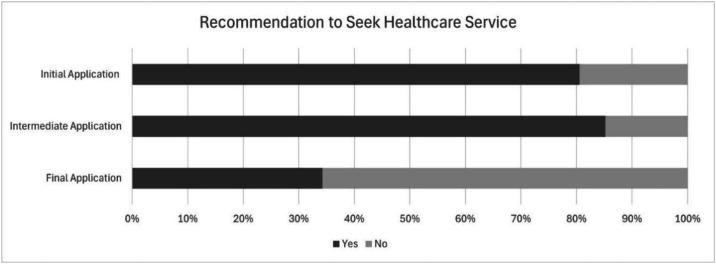
Recommendation to seek healthcare services.

The frequency with which the chatbot advised users to seek advice from a healthcare professional was also analyzed. In total, it recommended this action in only 66.6% of the responses provided (n = 216). In the first and second applications, this recommendation appeared in 80.6% and 85.2% of the cases, respectively, but there was a sharp decline in the third application, with only 34.3%, as shown in Figure 2. Analysis by question category revealed the following frequency of referral recommendation: definitions and concepts (71.2%), most frequent causes (48.6%), diagnostic methods (57.4%), therapeutics (82.5%), and disease or treatment repercussions (80.9%).

## Discussion

The study showed that the chatbot demonstrated an overall satisfactory performance, with 96.9% of responses rated as at least partially correct (scores 3–6), and a mode score of 6 indicating that many responses exceeded the reference answers provided by the specialists. However, it is important to note that this threshold includes scores of 3 (“correct but incomplete”) and 4 (“correct and complete but lacking clarity or appropriateness of language”), which may still be insufficient in a medical information context. Applying stricter thresholds, 77.2% of responses scored 4–6 and 46.3% scored 5–6. These results highlight that, while overall performance was encouraging, a non-trivial proportion of responses may be incomplete or unclear for patients and families.These findings suggest that ChatGPT possesses a strong grasp of theoretical and conceptual aspects of pediatric growth disorders. However, 10 answers were evaluated as incorrect (scores 1 and 2), and although this number is low, representing only 3.1% of the sample, it indicates a potential risk to the user, who must always be attentive to the tool’s errors and hallucinations.

When analyzing each question category individually, we observed significant variation. The chatbot performed best when answering questions related to definitions and concepts, common causes, and disease or treatment repercussions, areas requiring primarily factual recall, whereas accuracy declined in diagnostic and therapeutic domains. These latter categories require more advanced medical knowledge and specific information related to Brazil, such as the brands and formulations of medications available in the country.

**Figure 3:**
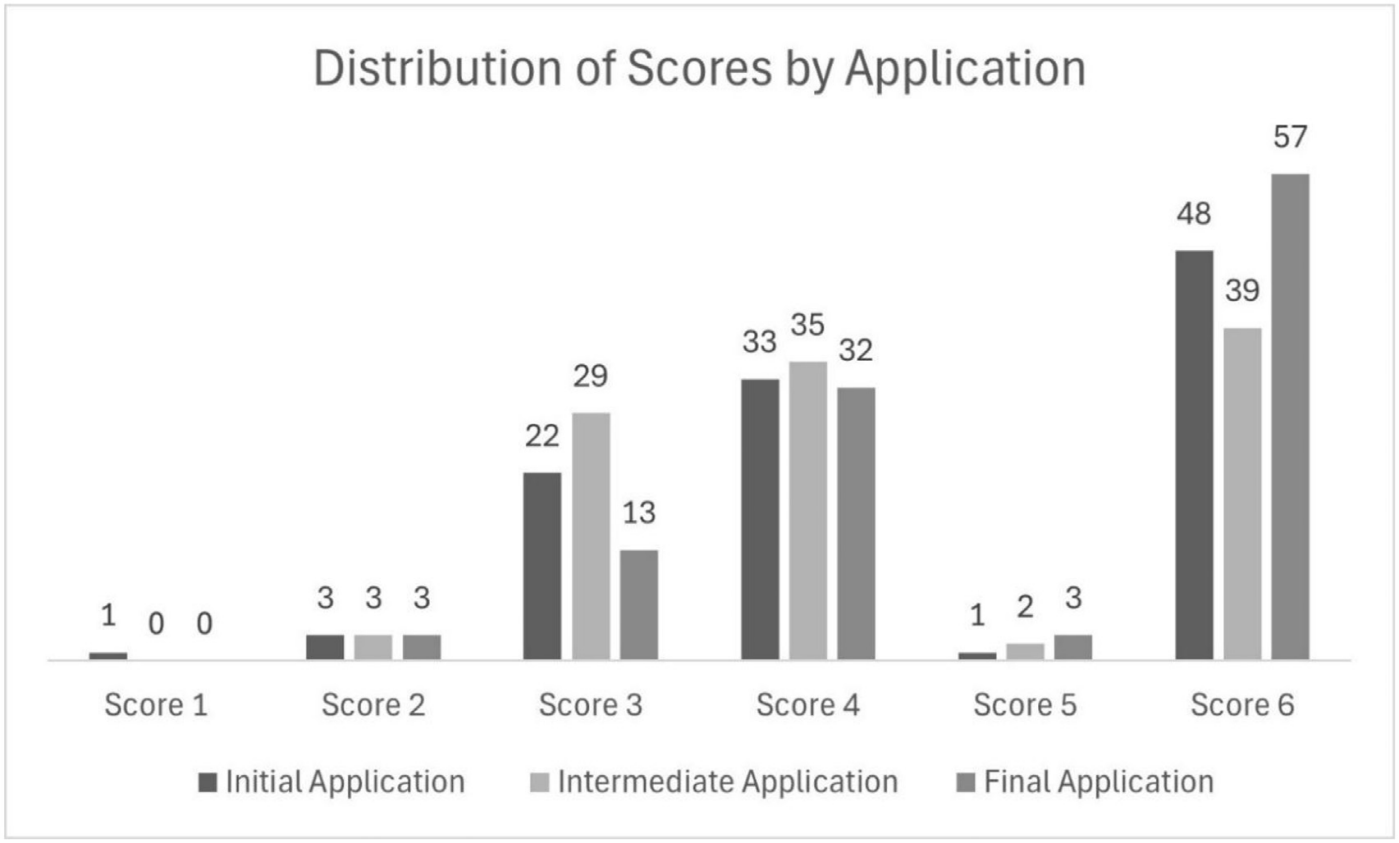
Distribution of scores by application.

No significant differences were observed between the first and second evaluations, both conducted using GPT-4. In contrast, GPT-4o demonstrated a higher proportion of improved responses, supporting the notion that periodic model updates resulted in greater agreement between the answers of the program and those of the pediatric endocrinology specialists. This relationship is illustrated in Figure 3.

Our results are consistent with the growing body of evidence on LLM performance in medical and health-related contexts. Thirunavukarasu et al. [5] synthesized the evidence on LLMs in medicine, noting that models such as GPT-4 demonstrate strong performance in factual recall tasks but face important limitations in clinical reasoning and context-dependent decision-making, a pattern closely mirrored in our findings. In standardized medical examinations, Kung et al. [6] demonstrated that ChatGPT (GPT-3.5) performed at or near the passing threshold (∼60%), while GPT-4 achieved approximately 81% accuracy, outperforming the average scores of medical students, highlighting meaningful medical knowledge acquisition alongside relevant limitations. In the more specialized context of pediatric endocrinology, Ongen et al. [9] found that LLMs including ChatGPT provided generally accurate and comprehensible answers to patient questions about type 1 diabetes in children, though performance declined with increasing question complexity. Liao et al. [10] assessed ChatGPT across four pediatric endocrine and metabolic conditions, including short stature, and reported superior performance for clinician-oriented questions, with lower accuracy in contextually nuanced clinical reasoning, particularly in diagnostics and therapeutics. Our study extends these findings by evaluating ChatGPT longitudinally across three time points and two model versions, enabling assessment of both accuracy and model evolution over time, with a direct comparison against expert-formulated reference answers.

The lower accuracy observed in the diagnostic methods and therapeutics domains is consistent with findings across the LLM literature and likely reflects multiple converging factors. First, these domains require the integration of patient-specific clinical context, sequential reasoning, and familiarity with locally applicable protocols, competencies that fundamentally exceed the scope of a generative language model trained on static text corpora. Second, therapeutic decision-making in pediatric endocrinology, such as the selection of GH formulations, dosing regimens, and monitoring protocols, depends on information that is both country-specific and temporally dynamic, including approved indications, available brands, and formulary details applicable to Brazil [14]. Such granular, regionally specific knowledge may not be uniformly or accurately represented in the model’s training data. Third, LLMs are known to produce “hallucinations”, plausible but factually incorrect responses, which pose disproportionate risks in high-stakes domains such as drug dosing and treatment selection [5]. These limitations reinforce the importance of caution when ChatGPT is consulted for therapeutic guidance, particularly in a pediatric context where inappropriate management can lead to irreversible consequences on growth and development.

The sharp decline in referral-to-specialist recommendations between GPT-4 (80.6% and 85.2% in the first and second applications) and GPT-4o (34.3% in the third application) represents not merely a quantitative change but a qualitative shift in the model’s behavior with direct implications for patient safety. Ayers et al. [15], in a landmark study comparing ChatGPT and physician responses to patient questions posted on a public social media forum, demonstrated that ChatGPT produced high-quality and empathetic answers; however, referral behavior was not the primary outcome evaluated. The reduction we observed in GPT-4o is consistent with a broader trend in successive LLM updates: as models are refined to deliver more comprehensive and self-contained responses, the recommendation to seek professional care may be progressively deprioritized, whether as a result of reinforcement learning from human feedback optimizing for user satisfaction, or of deliberate model alignment choices. In the specific context of pediatric growth disorders, where timely diagnosis of treatable conditions such as GH deficiency, hypothyroidism, or Turner syndrome is directly linked to final height outcomes and long-term well-being, any systematic reduction in AI-generated referral recommendations constitutes a significant safety concern. This finding underscores the need for ongoing post-deployment monitoring of LLM behavior, not only for factual accuracy, but also for safety-relevant output characteristics such as the appropriate encouragement of professional consultation.

From a clinical practice standpoint, the overall pattern of results suggests that ChatGPT may serve a useful complementary role in patient and family education, particularly for conceptual and descriptive questions related to growth percentiles, familial patterns, common causes, and the psychosocial repercussions of growth disorders and their treatment. However, it should not be used as a primary source for diagnostic or therapeutic guidance. Healthcare providers should be aware that patients and families may consult ChatGPT before or between appointments, and that the information they receive may be partially correct but potentially incomplete, overly general, or lacking the specificity required for individual clinical decision-making. Additionally, in a context such as Brazil, where limited access to pediatric endocrinology specialists is well documented [11], there is a particular risk that AI-generated information may substitute, rather than complement, professional care. Clinical institutions should consider providing clear guidance on the appropriate and safe use of AI tools as an adjunct to medical consultations, and future versions of LLMs dedicated to health applications should incorporate explicit and consistent referral-to-care recommendations, regardless of model updates [5].

This study has several limitations that should be noted. First, the questionnaire included a limited number of questions based on what the authors considered the main issues related to the topic, which may restrict generalizability. Second, the scoring relied on subjective interpretation of the responses, which may represent a bias. Finally, the test was conducted using ChatGPT versions 4.0 and 4o and therefore does not evaluate other versions of the tool.

## Conclusion

ChatGPT demonstrated high overall reliability, with low error rates. The degree of agreement between pediatric endocrinology specialists and the chatbot was high, particularly in less specific topics such as definitions and concepts, common causes, and disease or treatment repercussions. Performance was lower in subjects requiring more advanced medical knowledge. Regarding the tool’s evolution over time, while short-term effects were not evident, the chatbot showed significant improvement following the introduction of updated versions of the program. The sharp decline in recommendations to seek professional healthcare highlights a potential risk to users’ health.

The advent of artificial intelligence and freely accessible chatbots is, considered by many to be a major advancement. However, although ChatGPT holds promise as an educational and supportive tool in medical practice, caution is still required to ensure its safe and appropriate use. Although the responses generated by the tool generally received high scores, further development is necessary before its use can be broadly integrated into clinical practice. Medical professionals and patients seeking information through ChatGPT should be aware of its limitations and actively verify whether the information generated by AI aligns with that provided by reliable and authoritative sources.

## Authors’ contributions

RCA: Conceptualization, Data curation, Formal analysis, Investigation, Methodology, Project administration, Visualization, Writing – original draft, Writing – review & editing. CAL: Resources, Software, Supervision, Validation. All authors have approved the final article.

## Funding

This study was supported by the Brazilian National Council for Scientific and Technological Development (CNPq) through an Undergraduate Research Scholarship.

## Data availability statement

The data that support the findings of this study are available from the corresponding author upon reasonable request.

## Ethics Statement

Not applicable.

## Acknowledgement

The authors have no acknowledgments to report.

## Conflicts of interest

Nothing to declare.

## Appendix Supplementary materials

Image, application 1

## References

[1] Achiam J., Adler S., Agarwal S., Ahmad L., Akkaya I., Aleman F.L. (2023). GPT-4 technical report. arXiv [Preprint].

[2] Walsh S. (2023). Timeline of ChatGPT updates and key events [Internet]. Search Engine J..

[3] Similarweb. Website performance [Internet]. 2023 [cited 2023 May 16]. Available from:https://pro.similarweb.com/#/digitalsuite/websiteanalysis/overview/websiteperformance/*/999/3m?webSource=Total&key=chat.openai.com

[4] OpenAI. GPT-4o system card [Internet]. 2024 [cited 2024 May 16]. Available from: https://openai.com/index/gpt-4o-system-card/

[5] Thirunavukarasu A.J., Ting D.S.J., Elangovan K., Gutierrez L., Tan T.F., Ting D.S.W (2023). Large language models in medicine. Nat Med.

[6] Kung T.H., Cheatham M., Medenilla A. (2023 Feb). Performance of ChatGPT on USMLE: potential for AI-assisted medical education using large language models. PLOS Digit Health.

[7] Eysenbach G. (2003). The impact of the internet on cancer outcomes. CA Cancer J Clin.

[8] Hill M.G., Sim M., Mills B. (2020). The quality of diagnosis and triage advice provided by free online symptom checkers and apps in Australia. Med J Aust.

[9] Ongen Y.D., Aydin A.I., Atak M., Ozen S., Altinok Y.A., Darcan S. (2025). Performance of several large language models when answering common patient questions about type 1 diabetes in children: accuracy, comprehensibility and practicality. BMC Pediatr.

[10] Liao Y., Li X., Chen Y., Nie M., Li S., Wu X. (2024). Screening/diagnosis of pediatric endocrine disorders through the artificial intelligence model in different language settings. Eur J Pediatr.

[11] Scheffer M. (2025). http://bvsms.saude.gov.br/bvs/publicacoes/demografia_medica_brasil_2025.pdf.

[12] Collett-Solberg P.F., Jorge A.A.L., Boguszewski M.C.S., Miller B.S., Choong C.S.Y., Cohen P. (2019). Growth hormone therapy in children; research and practice – a review. Growth Horm IGF Res.

[13] Monte O., Longui C.A., Calliari L.E., Kochi C. (2006).

[14] Guimarães M.M., Bordallo M.A.N., Cargnin K.R.N., Souza H.F., Monteiro C.B. (2024). Endocrinologia pediátrica: guia prático.

[15] Ayers J.W., Poliak A., Dredze M., Leas E.C., Zhu Z., Kelley J.B. (2023). Comparing physician and artificial intelligence chatbot responses to patient questions posted to a public social media forum. JAMA Intern Med.

