# Supplementary material for: Evaluation of ChatGPT’s reliability in answering questions about short stature and growth failure
Source: J Pediatr (Rio J). 2026 Jul 2;102(5):101577. doi: 10.1016/j.jped.2026.101577 (PMC13352365; doi:10.1016/j.jped.2026.101577)
Supplement: Supplementary file 1 [file mmc1.docx]

**JPED-D-26-00139**

**SUPPLEMENTARY MATERIAL**

**Questionnaire and Expected Answers**

**1. Definitions and Concepts**

**1.1. What does the height percentile represent**

What is the height percentile?

What does the height percentile represent?

What is the meaning of height percentile?

**Expected answer:** Percentile is a measure of relative frequency divided into 100 parts, capable of representing the general population. The height percentile allows comparison of an individual’s height with that of the population of the same age and sex. Saying that a child is at the 10th percentile, for example, means that among 100 children of the same age and sex, they are taller than 10 and, consequently, shorter than 90.

**1.2. How to define height as normal**

What is normal height?

How can one know if height is within the limits of normality?

How to define height as normal?

**Expected answer:** The main way to classify an individual’s height as normal is through percentile curves (height between P2.5 and P97.5 is considered normal), which correspond to standard deviations or Z-scores (between -2 and +2). To define normality, parental height (target height) and the proportions between body segments are also taken into consideration.

**1.3. What is target height**

What is target height?

What does target height mean?

How to calculate and interpret target height?

**Expected answer:** Target height represents the genetic inheritance associated with parental height. The calculation is performed according to the formula: paternal height + maternal height ±13 cm / 2. The 13 cm represent the difference between male and female percentiles at 20 years of age. For boys, 13 cm are added, and for girls, 13 cm are subtracted. Allowing a margin of error of 2 cm above and below, there will be a 70% concordance between final height and target height.

**1.4. What is final height**

What is final height?

How to define final height?

What does final height mean?

**Expected answer:** Final height is the height reached by an individual in adulthood, after the end of the growth process. It is definitively achieved after the disappearance of the growth cartilage of the long bones, with consequent fusion of the epiphyses.

**1.5. What is short stature**

What is short stature?

How to define short stature?

What does short stature mean?

**Expected answer:** Short stature is defined as any height below the 2.5th percentile (or less than −2 SD) for a given age and sex.

**1.6. What is deficient growth or growth delay**

What is deficient growth?

What does deficient growth mean?

How to define deficient growth?

**Expected answer:** For a child to maintain a stable height percentile, it is necessary that they grow at the same rate as the average of children of the same age and sex (50th percentile). Growth is considered deficient when the growth velocity persistently remains below the 50th percentile, therefore associated with a progressive drop in height percentile.

**1.7. What is prediction of final height**

What is prediction of final height?

What does prediction of final height mean?

What does the prediction of final height represent?

**Expected answer:** It is an estimate of the adult height of a child based on current height, bone maturation (bone age), and pubertal stage. It usually employs tables of height fractions still to be achieved, according to the degree of bone maturation or pubertal stage.

**1.8. What is disproportionate short stature**

What is disproportionate short stature?

When can short stature be considered disproportionate?

What does disproportionate short stature mean?

**Expected answer:** Disproportionate short stature is a condition in which the individual has a total height lower than expected for age and sex, and in addition, the ratio between upper and lower body segments does not follow the expected proportions (for example, an individual with a trunk of normal size and limbs shorter than expected).

**2. Most Frequent Causes**

**2.1. What are the most frequent causes of short stature**

What are the most frequent causes of short stature?

What are the most common reasons behind short stature?

What are the most recurrent factors leading to short stature?

**Expected answer:** Short stature can be caused by endocrine or non-endocrine factors. Among the most common endocrine causes are growth hormone (GH) deficiency and hypothyroidism. Among the non-endocrine causes, the main ones are protein-calorie malnutrition, genetic alterations, and chronic systemic diseases (renal, gastrointestinal, cardiac, metabolic, etc.).

**2.2. What is familial short stature**

What is familial short stature?

What is familial short height?

What does familial short stature mean?

**Expected answer:** Familial short stature occurs when the child has short stature but follows the genetic pattern of parental height. In such cases, the growth rate is normal and bone age is compatible with chronological age. It can occur in the absence of disease or be associated with hereditary conditions present in one or both parents.

**2.3. When to say that a child was born small**

When to say that a child was born small?

Under what circumstances can a newborn be considered small at birth?

When is it appropriate to say that a baby was born small?

**Expected answer:** Birth size is related to gestational age, therefore, to assess the length of a newborn, percentile or Z-score curves should be used, correcting for gestational age. There is also a difference between sexes, boys being slightly larger than girls. For girls born at term, the average length is 50 cm, and being considered small for gestational age (SGA) corresponds to a length < 46 cm (3rd percentile). For boys, the average birth length is 51 cm, and SGA is considered when the boy is born < 47 cm (3rd percentile).

**2.4. What are the constitutional variants of growth and puberty**

What are the constitutional variants of growth and puberty?

What are the natural variations in growth and puberty?

What are the determinants of constitutional diversity in the process of growth and puberty?

**Expected answer:** Constitutional variants correspond to variations in the age at which the growth spurt and pubertal development occur. Those who mature within two years preceding the median age observed in the general population are known as early maturers (constitutional advancement of growth and puberty, CAGP), and those who mature within two years following the median age are late maturers (constitutional delay of growth and puberty, CDGP). Individuals with CDGP grow more slowly after 3–4 years of age until the onset of puberty, proportionally delaying pubertal onset, which increases total growth time and compensates for final height. However, in 20–30 % of individuals, pubertal delay is not proportional to the previous slowing of growth, and therefore final height may be compromised.

**2.5. What are the most frequent endocrine causes of short stature**

What are the most frequent endocrine causes of short stature?

What are the most frequent endocrine factors in short stature?

What are the most prevalent hormonal causes of short stature?

**Expected answer:** The most frequent endocrine causes of short stature are GH deficiency, hypothyroidism, and deficiency of sex hormones.

**2.6. What are the causes of growth hormone deficiency (GHD)**

What are the causes of growth hormone deficiency (GHD)?

How can the lack of growth hormone (GHD) in the organism be explained?

What are the reasons that may result in growth hormone deficiency (GHD)?

**Expected answer:** Growth hormone deficiency (GHD) can be congenital or acquired. In about half of GHD patients, there is a functional failure of GH secretion without an evident organic cause. However, in the other half, pituitary MRI may reveal anatomical alterations such as midline formation defects, reduced anterior pituitary (adenohypophysis), and ectopic posterior pituitary (neurohypophysis); presence of tumours in the region (most commonly craniopharyngioma); hydrocephalus, etc. Patients with cleft lip and palate require special attention to investigate GHD.

**2.7. What are the causes of disproportionate short stature**

What are the causes of disproportionate short stature?

What factors can lead to disproportionate short stature?

What are the origins of short stature that does not follow expected body proportions?

**Expected answer:** Disproportionate short stature occurs when the individual has a height below that expected for their age and sex, and in addition, the proportion between upper and lower body segments is not adequate, with the lower limbs generally being shorter than the trunk. The main causes of this condition are skeletal dysplasias such as achondroplasia and hypochondroplasia, genetic syndromes such as Turner syndrome, inborn errors of metabolism with storage diseases such as mucopolysaccharidoses, and endocrine alterations such as hypothyroidism and pseudohypoparathyroidism.

**2.8. When to consider that short stature is caused by Turner syndrome**

When to consider that short stature is caused by Turner syndrome?

What indicators suggest the possibility that short stature is caused by Turner syndrome?

Under what circumstances should Turner syndrome be considered as a possible cause of short stature?

**Expected answer:** Turner syndrome is a genetic condition that affects individuals with a female phenotype and is caused by total or partial absence of one X chromosome. It is possible to suspect that short stature is caused by this syndrome when other syndromic features are present, such as webbed neck, low-set ears, broad chest, and delayed pubertal development. Regardless of additional clinical findings, any girl with unexplained short stature should be evaluated to rule out Turner syndrome.

**3. Diagnostic Methods**

**3.1. What are the criteria for diagnosing familial short stature**

What are the criteria for diagnosing familial short stature?

What indicators suggest the diagnosis of familial short stature?

What are the clinical parameters considered when diagnosing hereditary short stature?

**Expected answer:** To diagnose familial short stature, it is necessary to assess the height of the child’s father and mother, calculate the familial target height, and demonstrate that it falls below the lower limits of the general population, that is, target height below 150 cm for women and 163 cm for men. In addition, the patient’s growth should show a constant pattern without deceleration and pubertal development starting at a normal time. Finally, chronic systemic diseases, genetic syndromes, and hormonal deficiencies must be excluded. An important limitation in the application of diagnostic criteria appears when there is height discordance between the parents, that is, one parent is short and the other is not. In this situation, the genetic determinant causing short stature may originate from the shorter parent.

**3.2. What is bone age**

What is bone age?

What is the meaning of bone age?

What does bone age represent?

**Expected answer:** Bone age represents a stage of bone maturation, meaning the point at which skeletal development has reached up to adulthood. This criterion uses radiographs (usually of the hands and wrists) capable of identifying the number of ossified centres, the shape of each nucleus, and the pattern of closure of the growth plates present in the metacarpals and phalanges. Bone age defines the bone maturation pattern of an individual, indicating the chronological age to which this pattern would correspond under normal conditions.

**3.3. How to use bone age in the diagnosis of short stature**

How to use bone age in the diagnosis of short stature?

How is bone age used in the evaluation of children with short stature?

What is the role of bone age in diagnosing short stature?

**Expected answer:** By comparing a child’s chronological age with their bone age, it is possible to recognise whether there is a delay or advancement in skeletal development. Some clinical situations are associated with delayed bone age (malnutrition, chronic systemic diseases, chronic use of glucocorticoids, hypothyroidism, etc.), while others show advanced bone age (obesity, precocious puberty, exogenous use of androgens or oestrogens). Based on the concept that epiphyseal closure determines the end of growth in an individual, it is possible to assess the growth potential remaining in the child. In other words, the more advanced the bone age, the lower the remaining growth potential. This aspect allows us to infer, from current height and bone age, the residual growth and therefore the predicted final height of a child.

**3.4. What are the criteria for diagnosing growth hormone deficiency (GH deficiency)**

What are the criteria for diagnosing growth hormone deficiency (GH deficiency)?

What are the parameters used to diagnose GH deficiency?

What clinical indicators are considered when diagnosing GH deficiency?

**Expected answer:** To diagnose GH deficiency in a patient with reduced growth velocity or short stature, a complete clinical evaluation is required (growth history, family pattern, identification of syndromic signs or other diseases that may determine growth loss), assessment of bone age, and identification of loss of predicted final height. Hypothyroidism must be excluded and the insulin-like growth factor type 1 (IGF-1) level quantified. Only then should stimulation tests for GH release (clonidine, glucagon, insulin) be requested.

**3.5. Should pituitary magnetic resonance imaging (MRI) be performed during investigation of GH deficiency**

Should pituitary MRI be performed during investigation of GH deficiency?

Is it recommended to order a pituitary MRI when investigating the origin of GH deficiency?

When is it appropriate to consider performing a pituitary MRI as part of GH deficiency evaluation?

**Expected answer:** Yes, pituitary MRI should be performed during the investigation, along with clinical assessment and family history, and exclusion of other chronic diseases. MRI is usually carried out after confirmation of GH deficiency through stimulation tests. In situations where the clinical picture is suggestive and the child is older than six years, a fast and simplified MRI protocol (FAST1) can be performed without anaesthesia and before the stimulation tests. This examination can identify, in up to half of patients, pituitary region abnormalities characteristic of hormonal deficiency, which reduces the need for stimulation testing.

**3.6. What tests should be performed to confirm the hypothesis of Turner syndrome**

What tests should be performed to confirm the hypothesis of Turner syndrome?

Which tests are necessary to corroborate the suspicion of Turner syndrome?

What clinical examinations should be performed to confirm the hypothesis of Turner syndrome?

**Expected answer:** Turner syndrome is one of the causes of growth reduction and short stature in girls. To diagnose Turner syndrome, karyotyping is used, which in this condition identifies the complete or partial loss of one of the X chromosomes, with the most characteristic pattern being 45,X, indicating total absence of one X chromosome.

**4. Therapeutics**

**4.1. What is the usefulness of physical exercise in height gain**

What is the usefulness of physical exercise in height gain?

In what way does physical activity influence height gain?

How does the practice of physical exercise contribute to height increase?

**Expected answer:** Height is determined by several factors, the main one being genetics. However, normal growth depends critically on a qualitatively and quantitatively adequate diet, regular sleep pattern, and healthy and regular physical activity. The lack or excess of these factors generally has a negative influence on growth. Children and adolescents subjected to intense training (more than 3–4 hours per day) may have impaired growth and final height, as well as delayed puberty.

**4.2. What dietary pattern is recommended to accelerate height gain**

What dietary pattern is recommended to accelerate height gain?

What are the dietary recommendations that help promote height gain?

What are the dietary guidelines advised to stimulate height gain?

**Expected answer:** Ideal growth is supported by a balanced diet, without qualitative or quantitative excesses or deficiencies. This dietary pattern must be adjusted to the stage of development and the level of physical activity of each individual. It is recommended that the diet include adequate amounts of proteins, minerals, and vitamins, promoting bone and muscle mass gain and meeting the energy expenditure adjusted to the degree of physical activity.

**4.3. When to use growth hormone (GH) in the treatment of short stature**

When to use growth hormone (GH) in the treatment of short stature?

What are the indications for using growth hormone (GH) in the treatment of short stature?

In which circumstances is it appropriate to use GH in the treatment of children with short stature?

**Expected answer:** Replacement treatment with GH is indicated for short stature caused by pituitary GH production deficiency. Currently, several approved indications for GH use exist, such as children born small for gestational age, chronic renal insufficiency, idiopathic short stature (familial, constitutional delay without catch-up, idiopathic per se, etc.), Turner syndrome, Noonan syndrome, and Prader–Willi syndrome, among others.

**4.4. What GH dose can be used**

What GH dose can be used?

What are the recommendations regarding the amount of GH that can be used?

What is the GH dose that can be prescribed?

**Expected answer:** The replacement dose of recombinant GH is 0.033 mg/kg/day (0.1 IU/kg/day), subcutaneously, once daily. Specifically for GH-deficient patients, in Brazil there is approval for weekly GH formulations such as Somapacitan (Sogroya, Novo Nordisk) at 0.16 mg/kg/week and Somatrogon (Genryzon, Pfizer) at 0.66 mg/kg/week. For non-GH-deficient patients, the dose is 0.05 mg/kg/day (0.15 IU/kg/day). For these cases, the approved Somapacitan dose is 0.24 mg/kg/week.

**4.5. Which brands and presentations of growth hormone are available in Brazil**

Which brands and presentations of growth hormone are available in Brazil?

What are the brands and presentations of growth hormone available in the Brazilian market?

In Brazil, what options of GH brands and presentations are available?

**Expected answer:** In Brazil, the following recombinant GH brands are approved:

**Daily use:** Bergamo (Hormotrop – syringe injection), Cristália (Criscy – syringe injection and also pen presentation), Merck Serono (Saizen Easypod – electronic self-injector, and Saizen Aluetta – pen), Novo Nordisk (Norditropin – pen), Pfizer (Genotropin – pen), Sandoz (Omnitrope – pen).

**Weekly use:** Novo Nordisk (Sogroya – pen), Pfizer (Genryzon – pen).

**4.6. What are the differences between daily-use GH and weekly-use GH**

What are the differences between daily-use GH and weekly-use GH?

What features distinguish daily GH from weekly GH?

Describe the particularities of daily-use GH compared with weekly-use GH.

**Expected answer:** Since 1985, the GH approved and available for the treatment of short stature has been synthetic GH obtained through genetic engineering techniques, known as recombinant human GH (rhGH). It has the same sequence as endogenous human pituitary GH (22 kDa). Currently, two new long-acting rhGH formulations are available, administered once a week:

(i) **Somatrogon (Genryzon):** adds to the GH molecule one copy of the C-terminal peptide (CTP) of the beta chain of human chorionic gonadotrophin in the N-terminal region and two CTP copies in the C-terminal region. The CTP and glycosylation domains are responsible for Somatrogon’s longer half-life.

(ii) **Somapacitan (Sogroya):** presents a single substitution in the main amino acid structure (L101C), to which an albumin-binding component is attached. The albumin-binding component (side chain) consists of a fatty acid component and a hydrophilic spacer.

**4.7. Are there alternatives to GH use in the treatment of children with short stature**

Are there alternatives to GH use in the treatment of children with short stature?

Besides GH, are there other treatment options for children with short stature?

Are there possible substitutes for GH use to treat short stature in children?

**Expected answer:** Non-hormonal drugs tested in the 1970s and 1980s, including trace elements and non-hormonal stimulants of GH release, proved ineffective in changing growth rate or final height. General care in managing children with short stature includes an adequate diet in both qualitative and quantitative aspects, as well as regular physical activity appropriate for healthy children. GH deficiency should be treated with replacement GH administration. Other causes of short stature also have approved GH indications, such as Turner syndrome, small for gestational age, chronic renal insufficiency, Noonan syndrome, Prader–Willi syndrome, and idiopathic short stature.

**5. Repercussions of Treatment**

**5.1. What would be the psychosocial benefits of using growth hormone**

What would be the psychosocial benefits for children with short stature after the use of growth hormone?

What psychosocial advantages can be expected with growth hormone treatment?

What possible psychosocial improvements might patients experience when using growth hormone?

**Expected answer:** The increase in height promoted by treatment with growth hormone (GH) can improve children’s self-esteem, avoiding situations such as bullying, increasing confidence and well-being, and reducing social stigma. Indirectly, there may be better participation in group activities, such as recreational physical activities.

**5.2. What additional benefits can GH offer besides promoting growth**

What additional benefits can GH offer besides promoting growth?

In addition to stimulating growth, what other advantages can GH provide?

What are the possible additional gains resulting from GH use besides growth?

**Expected answer:** In addition to promoting growth, GH use increases bone mass and muscular contractile efficiency, thus enhancing energy expenditure and performance in motor activities. It also acts on adipose tissue, facilitating lipolysis.

**5.3. What are the main side effects resulting from the use of growth hormone**

What are the main side effects resulting from the use of growth hormone (GH)?

Describe the most frequent side effects resulting from the use of GH.

What are the main adverse effects that patients may experience when using GH?

**Expected answer:** GH use tends to cause water and salt retention at the beginning of treatment and may rarely cause signs of benign intracranial hypertension (usually reversible upon discontinuation of the medication), insulin resistance, as well as local inflammatory reactions. Transient oedema of the extremities may also occur. Gynaecomastia, accentuation of scoliosis, tonsillar hypertrophy, and slippage of the femoral head have also been reported.

**5.4. What are the contraindications to GH use**

What are the contraindications to GH use?

Describe the contraindications for using growth hormone (GH).

Under what circumstances is GH use contraindicated?

**Expected answer:** GH should not be used in patients with active neoplasms (tumours), patients with uncontrolled growth of benign intracranial tumours, in those with decompensated diabetes or diabetic retinopathy, and in acutely and critically ill patients due to complications of surgical procedures, severe trauma, or acute respiratory failure.

**5.5. Is there a cause–effect relationship between GH use and tumor development**

Is there a direct connection between GH use and tumor formation?

Is GH use associated with a risk of developing tumors?

Is there evidence that GH may play a causal role in tumor development?

**Expected answer:** There is no evidence of a direct cause–effect relationship between GH use and the appearance of tumours of any type. Individuals who have had previous tumours or received radiotherapy have a higher risk of developing new tumours and therefore should not receive GH.

**5.6. What height gain is expected in those who use GH**

What height gain is expected in those who use GH?

What is the average increase in final height expected for those treated with GH?

What additional height increase is expected in individuals using GH?

**Expected answer:** The growth response to rhGH is highly variable. Predictors of a greater height response to rhGH treatment include: the underlying disease motivating treatment, lower chronological age, and greater height deviation from target height (genetic potential) at therapy initiation. The goal of treatment is to achieve a growth velocity percentile always above that normally observed for the patient’s age and sex. The greatest gains are observed in GH-deficient patients treated early. An intermediate gain of 7 cm in final height was observed in patients with idiopathic short stature treated with GH at a dose of 0.15 IU/kg/day (0.05 mg/kg/day). The poorest responses are obtained in the treatment of primary cartilage growth disorders and in patients who began treatment late during puberty.

**5.7. What factors interfere with height gain during GH treatment**

What factors interfere with height gain during GH treatment?

What elements impact growth during GH therapy?

What variables may affect height gain during GH use?

**Expected answer:** Determining factors include chronological age at GH initiation (better the younger the age), GH dose (better the higher the dose), and severity of short stature in relation to familial target height (better response the further below the familial target height). These factors are especially relevant in the first year of treatment. Long-term therapeutic response is influenced by the same factors, together with familial target height (better response the higher the familial target height). Less promising results have been observed in patients with idiopathic short stature in the midst of puberty.
